# Secondary Diffuse Large B‐Cell Lymphoma After Sequential Tyrosine Kinase Inhibitor Therapy in Chronic Myeloid Leukemia: A Case Report

**DOI:** 10.1155/crh/4762512

**Published:** 2026-03-31

**Authors:** Zaineb Mlayah, Kmar Mrad, Inès Ben Rekaya, Inaam Bizid, Nader Slama, Mohamed Adnène Laatiri, Sarra Boukhris

**Affiliations:** ^1^ Hematology Department, Fattouma Bourguiba University Hospital, Monastir, Tunisia

**Keywords:** case report, chronic myeloid leukemia, diffuse large B-cell lymphoma, imatinib, nilotinib, secondary malignancy, tyrosine kinase inhibitor

## Abstract

**Background:**

Tyrosine kinase inhibitors (TKIs) targeting the BCR::ABL1 fusion protein have revolutionized the treatment of chronic myeloid leukemia (CML), transforming it into a chronic and manageable disease with markedly improved survival. However, prolonged TKI exposure has been associated with rare hematological complications, including secondary lymphoid malignancies.

**Case Presentation:**

We describe a 39‐year‐old woman with CML who developed diffuse large B‐cell lymphoma (DLBCL) after long‐term nilotinib therapy. She had previously received imatinib and dasatinib, both discontinued due to intolerance and cytogenetic failure. Five years after initiating nilotinib, she presented with gastric pain and right cervical lymphadenopathy. Histopathological examination confirmed a nongerminal center B‐cell (non‐GCB) subtype DLBCL (CD20+, MUM1+, BCL6+, Ki‐67: 80%). The patient received eight cycles of R‐CHOP, achieving complete remission. At the most recent follow‐up, she maintained a major molecular response for CML and complete remission for lymphoma.

**Conclusion:**

This case highlights a rare occurrence of DLBCL following long‐term TKI therapy for CML. Although a direct causal relationship remains unproven, cumulative drug exposure, immune dysregulation, and genomic instability may contribute to lymphomagenesis. Continuous long‐term surveillance is essential for early detection and management of secondary malignancies in patients receiving chronic TKI therapy.

## 1. Introduction

Chronic myeloid leukemia (CML) is a chronic myeloproliferative neoplasm that primarily affects the granulomonocytic lineage and typically occurs in adults, with a median age of 63 years.

It is characterized by the presence of the Philadelphia (Ph) chromosome, or more precisely, der(22)t(9; 22)(q34; q11), resulting from a reciprocal translocation between chromosomes 9 and 22 [1]. This abnormality leads to the formation of the BCR::ABL1 fusion gene, which encodes a constitutively active tyrosine kinase that promotes uncontrolled cell proliferation and inhibits apoptosis, the key driver of CML pathogenesis [2, 3].

CML progresses through three clinical phases: chronic, accelerated, and blast crisis. Effective treatment can maintain most patients in the chronic phase for many years [4].

Understanding the molecular mechanism of BCR::ABL1‐driven leukemogenesis has led to the development of tyrosine kinase inhibitors (TKIs), with imatinib being the first‐generation TKI designed through rational drug discovery [5]. The main therapeutic goals in chronic‐phase CML are to prolong survival, improve quality of life, and prevent transformation to the blast phase.

While imatinib remains the most cost‐effective first‐line treatment [6], other TKIs, dasatinib, nilotinib, and bosutinib (second generation) and ponatinib and olverembatinib (third generation), have been developed to overcome resistance or intolerance [6]. Allogeneic stem cell transplantation (allo‐SCT) remains an option for patients with multi‐TKI−resistant disease or intolerance, although its use is limited by age and donor availability [6].

The advent of TKIs has transformed CML into a manageable and often long‐term controllable disease. However, concerns persist regarding their long‐term safety, particularly the risk of secondary malignancies. This has also prompted the exploration of treatment‐free remission (TFR), discontinuing TKI therapy after achieving a deep and sustained molecular response for at least 2 years [7–9]. Unfortunately, in many developing countries, such as Tunisia, limited access to molecular monitoring often restricts the implementation of TFR.

Secondary malignancies associated with TKI therapy have been reported in approximately 3.6%–4.5% of patients, with secondary lymphomas representing around 3.5% of these cases [10]. However, the occurrence of diffuse large B‐cell lymphoma (DLBCL) following long‐term TKI therapy remains infrequently observed, with only a few cases reported in the literature.

Here, we present a case of DLBCL arising after prolonged nilotinib exposure in a patient treated for CML, highlighting diagnostic challenges and therapeutic considerations in this uncommon clinical presentation.

## 2. Case Presentation

Mrs. A.C., a 39‐year‐old woman, has been followed since 2007 in the Clinical Hematology Department of the Maternity and Neonatology Center of Monastir, Tunisia, for CML.

Her past medical history included asthma treated with corticosteroids and a cholecystectomy performed in 1993.

At diagnosis, reverse transcriptase–polymerase chain reaction (RT‐PCR) confirmed the presence of the BCR::ABL1 b3a2 transcript, and conventional cytogenetic analysis revealed the Ph chromosome in all examined metaphases. The patient was initially treated with imatinib 400 mg/day. After 6 months, she exhibited cytogenetic and molecular failure, accompanied by adverse effects (muscle cramps, vomiting, lower‐limb edema, and severe thrombocytopenia with platelets persistently < 50 × 10^9^/L). Increasing the imatinib dose to 600 mg/day failed to achieve a cytogenetic response after 12 months, prompting a switch to a second‐generation TKI.

On June 28, 2010, dasatinib 100 mg/day was initiated. Two months later, the patient developed hemorrhoidal bleeding and worsening thrombocytopenia (platelets: 28 × 10^9^/L). A temporary dose reduction to 50 mg/day, combined with a 3‐week corticosteroid course, led to hematologic recovery.

Cytogenetic assessment at 5 months (November 2010) revealed persistence of the Ph chromosome in 60% of metaphases and emergence of Trisomy 8 in 40%. After 9 months, Trisomy 8 became predominant (50%), while the Ph chromosome remained detectable in 45% of metaphases. Given recurrent thrombocytopenia and suboptimal response, initiation of nilotinib was planned. Pending approval, dasatinib was maintained.

Nilotinib 800 mg/day was started on January 29, 2013. Subsequent cytogenetic studies revealed the disappearance of the Ph chromosome, but the persistence of Trisomy 8. A major cytogenetic response was achieved in 2016 (35% Ph + metaphases). However, in early 2017, cytogenetic relapse occurred (75% Ph + metaphases) with a BCR::ABL1 ratio of 25% in November 2017. Allo‐SCT was not feasible due to the absence of a compatible HLA donor, and nilotinib was continued at the same dose.

In September 2018, five years after initiating nilotinib, the patient presented with diffuse bone pain, epigastric pain, and a right jugulo−carotid lymphadenopathy. Upper gastrointestinal endoscopy revealed an ulcerated, budding tumor of the gastric antrum. Histopathological examination confirmed DLBCL, non‐germinal center B‐cell (non‐GCB) subtype, immunophenotypically CD20+, MUM1+, and BCL6+, with a Ki‐67 proliferation index of 80%.

*Helicobacter pylori* testing was not documented at the time of initial diagnosis. However, as this is a retrospective report, no additional testing could be performed to retrospectively confirm the *H. pylori* status at the time of lymphoma diagnosis.

The staging workup demonstrated a 70‐mm thickening of the antral wall and hepatosplenomegaly (liver 20 cm; spleen 13.5 cm) without bone marrow involvement. PET‐CT was unavailable in Tunisia at that time.

According to the International Prognostic Index (IPI), the patient received eight cycles of rituximab, cyclophosphamide, doxorubicin, vincristine, and prednisone (R‐CHOP21) between October 10, 2018, and April 22, 2019.

Interim imaging (CT scan, January 2019) showed stabilization of the antral wall thickening, and follow‐up endoscopy demonstrated complete remission with mild fundic gastritis. Posttreatment endoscopy in April 2019 confirmed the absence of residual lymphoma and moderate chronic gastritis. Follow‐up gastric biopsies performed in 2020 showed no evidence of *Helicobacter pylori* infection.

During nilotinib continuation, molecular monitoring in December 2019 revealed a BCR::ABL1 ratio of 3.9%. Subsequent surveillance, combining radiologic (CT) and endoscopic assessments, showed sustained remission up to July 2023. The most recent endoscopy (December 2020) confirmed continued complete remission, and molecular testing (October 2023) demonstrated a major molecular response (BCR::ABL1 = 0.018%).

## 3. Discussion

Our patient developed DLBCL following sequential exposure to three different TKIs, with lymphoma appearing 5 years after the initiation of nilotinib. This case highlights the uncommon but clinically significant association between long‐term TKI therapy and secondary lymphomas in patients with CML.

TKIs have revolutionized CML management, transforming it from a fatal disease into a chronic, manageable condition with markedly improved survival. However, their long‐term safety profile remains under scrutiny, particularly regarding the potential for secondary malignancies such as lymphomas [10]. Establishing a direct causal relationship between TKIs and lymphomagenesis is complex. Prolonged survival naturally increases the likelihood of a second hematological neoplasm, but additional factors, including TKI type, cumulative exposure, genetic susceptibility, and environmental influences, may also contribute [11].

Experimental data suggest that TKIs may exert off‐target immunosuppressive effects by inhibiting kinases involved in immune regulation, leading to impaired immune surveillance and a permissive microenvironment for oncogenic transformation [12]. Such prolonged immunomodulation could predispose to lymphoproliferative disorders, with non‐Hodgkin lymphoma being the most frequently described [13].

To date, nine well‐documented cases of secondary lymphoma developing during TKI therapy have been identified in the literature [10, 11, 14–20]. Reported lymphomas encompass various histological subtypes, with imatinib being the most frequently implicated agent. Data from North Africa remain scarce, with only one previously reported case from Tunisia [20]. Our report contributes to this limited regional experience. A comparative summary of these previously published cases, together with the present report, is provided in Table 1.

**TABLE 1:** Reported cases of TKI‐associated secondary lymphoma in patients with chronic myeloid leukemia.

Author (year)	Age/sex	Lymphoma subtype	TKI (s) exposure	Duration before lymphoma	TKI management	Lymphoma treatment	Outcome
Rodler et al. (2004)	65/M	Blastic mantle cell lymphoma	Imatinib	∼1 year	Discontinued	R‐CHOP	Death
Mihaylov et al. (2016)	53/F	Extranodal MALT lymphoma	Imatinib	7 years	Continued	CHOP	CR
Gajendra et al. (2019)	50/M	Classical Hodgkin lymphoma	Imatinib ⟶ dasatinib	10 years	NR	ABVD	CR
Domínguez‐Pinilla et al. (2019)	8/M	Pediatric‐type nodal follicular lymphoma	Imatinib ⟶ dasatinib	34 months (dasatinib)	Discontinued ⟶ switched to nilotinib	Observation	CR
Lee et al. (2020)	81/M	Lymphoplasmacytic lymphoma	Imatinib ⟶ dasatinib ⟶ radotinib	15 years	NR	R‐CHOP	PR
Takakuwa et al. (2021)	75/M	High‐grade B‐cell lymphoma	Imatinib ⟶ nilotinib ⟶ bosutinib	36 months (bosutinib)	Discontinued	DA‐EPOCH‐R	CR
Paczkowska et al. (2021)	64/M	Classical Hodgkin lymphoma	Imatinib ⟶ nilotinib ⟶ dasatinib	19 months (dasatinib)	Continued at reduced dose	AVD	CR
Geelani et al. (2022)	49/M	Follicular lymphoma (Grade 1)	Imatinib	6 years	Discontinued	Bendamustine + rituximab	CR
Kassar et al. (2023)	54/F	Diffuse large B‐cell lymphoma	Imatinib	9 years	Discontinued	R‐CHOP	NR
Present case	39/F	Diffuse large B‐cell lymphoma (non‐GCB), gastric	Imatinib ⟶ dasatinib ⟶ nilotinib	5 years (nilotinib)	Continued at reduced dose	R‐CHOP	CR

*Note:* LPL, lymphoplasmacytic lymphoma; R‐CHOP, rituximab, cyclophosphamide, doxorubicin, vincristine, and prednisone; CHOP, cyclophosphamide, doxorubicin, vincristine, and prednisone; ABVD, doxorubicin, bleomycin, vinblastine, and dacarbazine; AVD, doxorubicin, vinblastine, and dacarbazine; DA‐EPOCH‐R, dose‐adjusted etoposide, prednisone, vincristine, cyclophosphamide, and doxorubicin plus rituximab; non‐GCB, nongerminal center B‐cell–like subtype.

Abbreviations: CML, chronic myeloid leukemia; CR, complete remission; DLBCL, diffuse large B‐cell lymphoma; HGBCL, high‐grade B‐cell lymphoma; MALT, mucosa‐associated lymphoid tissue; NR, not reported; PR, partial remission; TKI, tyrosine kinase inhibitor.

In addition, several reports have described lymphoid proliferations in patients with CML during TKI therapy; however, these were excluded from the present analysis because the lymphoma was diagnosed concomitantly with CML, represented Richter transformation, corresponded to benign lymphoid hyperplasia, or lacked sufficient evidence to support a true secondary TKI‐associated malignancy [21–25]. To ensure methodological rigor, only cases in which lymphoma developed after established TKI exposure were retained.

Notably, previously reported cases of concurrent CML and lymphoma at initial diagnosis, Richter transformation, and dasatinib‐associated follicular lymphoid hyperplasia were not considered secondary TKI‐associated lymphomas.

The temporal relationship between TKI exposure and lymphoma onset is a critical consideration. In our patient, DLBCL developed after approximately 3 years of imatinib, 3 years of dasatinib, and 5 years of nilotinib exposure. This prolonged multi‐TKI exposure raises questions about the causal agent. A delayed oncogenic effect of earlier agents (imatinib or dasatinib) cannot be excluded, while nilotinib’s contribution remains uncertain given the lack of exclusive reports. A cumulative, multiagent effect appears plausible and has been described in only two prior cases [10, 18], making ours the third reported instance. This underscores the need to explore the potential cumulative toxicity of sequential TKIs on genomic stability.

Another distinctive feature of this case is the gastric localization of DLBCL, an exceedingly rare presentation in TKI‐associated lymphomas. Imatinib’s known affinity for gastric tissues, due to its inhibition of KIT and PDGFRA, key regulators of gastrointestinal homeostasis, could theoretically contribute to localized oncogenic stress [26]. The absence of similar data for dasatinib and nilotinib suggests that the gastric site may reflect a delayed effect of prior imatinib exposure rather than a direct consequence of nilotinib therapy. A similar case involving sequential imatinib–nilotinib exposure has been previously reported [24], supporting this hypothesis.

*Helicobacter pylori* infection is a well‐recognized etiological factor in primary gastric lymphomas, including some cases of DLBCL, particularly those arising from a preexisting MALT lymphoma. In our patient, *H. pylori* testing was not documented at the time of initial diagnosis, which represents a limitation of this retrospective case report. Although follow‐up biopsies performed after treatment did not demonstrate evidence of *H. pylori* infection, we cannot exclude the possibility of a prior undetected infection. Therefore, while a contributory role of *H. pylori* cannot be definitively ruled out, the prolonged and sequential exposure to multiple TKIs remains a clinically relevant factor to consider in this context.

Cytogenetically, the emergence of Trisomy 8 during treatment, absent at diagnosis, may indicate genomic instability secondary to chronic TKI exposure. Some studies suggest that sustained BCR::ABL1 suppression may interfere with DNA repair mechanisms, potentially facilitating the development of chromosomal aberrations [20]. Further research is needed to confirm these observations.

Managing concomitant CML and secondary lymphoma presents a therapeutic challenge. Optimal management requires balancing effective disease control against compounded toxicity. Standard lymphoma therapy remains effective in these patients, provided treatment is individualized. In our patient, R‐CHOP was administered successfully while continuing nilotinib at a reduced dose, achieving complete remission of both diseases. Reported outcomes in the literature are generally favorable [10, 19, 20]. Some clinicians advocate discontinuing TKIs during chemotherapy to mitigate cytopenias, whereas others maintain reduced doses for CML control.

The prognosis of lymphoma arising in the context of iatrogenic immunosuppression is generally favorable compared to that of lymphoma associated with primary immune deficiencies. Therefore, such cases are typically managed as de novo lymphomas, with consideration of discontinuing the immunosuppressive agent when possible [27].

Interestingly, certain TKIs have demonstrated antitumor activity in follicular lymphoma [23], complicating the causal interpretation. Thus, rather than direct oncogenesis, TKIs may act as immunomodulatory cofactors in genetically susceptible individuals. Establishing a definitive causal relationship remains difficult, and further mechanistic studies are warranted.

## 4. Conclusion

In summary, this case illustrates the rare occurrence of DLBCL following long‐term, sequential exposure to multiple TKIs in a patient with CML. Although isolated reports have described similar associations, a clear causal link between TKI therapy and secondary lymphomagenesis remains unproven.

Our observation underscores the importance of vigilant, long‐term follow‐up for CML patients receiving TKIs, particularly those exposed to multiple agents over extended periods. Continued molecular and clinical surveillance is essential to detect emerging complications early and to optimize patient outcomes.

Finally, despite the therapeutic complexity of concomitant CML and lymphoma, our case demonstrates that conventional immunochemotherapy remains an effective and well‐tolerated treatment strategy. Further collaborative studies are warranted to clarify the mechanisms involved and to guide management strategies, including potential TKI discontinuation in patients achieving deep and sustained molecular remission.

## Funding

No funding was received for this study.

## Ethics Statement

Informed consent from the patient and authorization for publishing the case were obtained.

## Consent

Please see the Ethics Statement.

## Conflicts of Interest

The authors declare no conflicts of interest.

## Data Availability

Data sharing is not applicable to this article as no datasets were generated or analyzed during the current study.
